# Supplementary material for: Sub-Clinical Effects of Outdoor Smoke in Affected Communities
Source: Int J Environ Res Public Health. 2021 Jan 28;18(3):1131. doi: 10.3390/ijerph18031131 (PMC7908479; doi:10.3390/ijerph18031131)
Supplement: Supplementary file 1 [file ijerph-18-01131-s001.pdf]

MONASH University
School of Public Health and Preventive Medicine

Health effects of smoke from planned burning

Baseline Questionnaires

Region:

Locality:

Date ☐☐☐☐☐☐

☐ **MALE** ☐ **FEMALE**

TO ANSWER THE QUESTIONS PLEASE CHOOSE THE APPROPRIATE BOX
IF YOU ARE UNSURE OF THE ANSWER PLEASE CHOOSE 'NO'

1. What is your date of birth? ☐☐ **DAY** ☐☐ **MONTH** ☐☐☐☐ **YEAR**

2. For how many years you have been resident of this region? Years Months

3. What was your **highest** level of education (please select only one)?

☐completed primary school education ☐completed secondary school education ☐completed trade certificate/apprenticeship ☐completed tertiary qualification (e.g. university degree)

4. Are you currently in paid employment? ☐NO ☐YES

If **Yes**, Please specify

Full time ☐

Part time ☐

Please state your **current** occupation ______________________

If **No,**

I have retired ☐

Please state your occupation **before** retirement ______________________

5. Have you been told by a **doctor** that you have any of the following conditions?

5.1 Asthma ☐NO ☐YES

If **yes**

a) How old were you when you had your first attack of asthma ☐☐ ***Years***
(If started as a baby please enter 1)

b) Have you had an attack of asthma in the last 12 months ☐NO ☐YES

c) Are you currently taking any medication (including inhalers, aerosols, or tablets) for asthma ☐NO ☐YES

5.2 Chronic Obstructive Pulmonary Disease/airway disease-(COPD) ☐NO ☐YES

5.3 Other respiratory disease ☐NO ☐YES

5.4 Angina ☐NO ☐YES

5.5 High blood pressure/hypertension ☐NO ☐YES

5.6 High Cholesterol ☐NO ☐YES

5.7 Heart attack (this includes a myocardial infarction or coronary event) ☐NO ☐YES

5.8 Heart failure ☐NO ☐YES

5.9 Irregular heart rhythm/Arrhythmia ☐NO ☐YES

5.10 Stroke (this includes a 'mini stroke'/TIA cerebrovascular accident) ☐NO ☐YES

5.11 Other heart disease ☐NO ☐YES

6 Do you have Diabetes? ☐NO ☐YES
If **yes**

Are you on any medications (oral tablets/ insulin injections)? ☐NO ☐YES

7 Do you have any Inflammatory Disease (e.g. Rheumatoid Arthritis, Psoriasis)? ☐NO ☐YES
If **yes,** please describe

---

8 Any other medical condition not mentioned above. Please describe?

---

9 During the past 6 weeks have you suffered from any of the following: cold, flu, throat or chest infection?

☐NO☐YES

If **yes** are you on any medication (e.g. antibiotics, anti-inflammatories)? ☐NO☐YES

10. Please list all **medications** you are currently taking (started in the last six weeks)?

11. Please list all **medications** you are taking on a <u>regular/on-going basis</u>

11. Do you currently **smoke**, or have you ever smoked for as long as a year?

NEVER SMOKED ☐

YES CURRENTLY SMOKING☐

YES, BUT STOPPED☐

If yes, please answer the questions below:

How old were you when you started smoking regularly? ☐☐ ***Years***

Have you smoked in the last month? ☐NO☐YES

How old were you when you last smoked? ☐☐ ***Years***

12. What is the main type of **home heating** you use (please tick)?

Electric ☐

Gas ☐

Hydronic ☐

Solar ☐

Wood heating - open fire place ☐

Wood heating – enclosed wood heater ☐

13 What type of **cooking burner** do you use?

Electric ☐

Gas ☐
